# Development and validation of a short dietary questionnaire for assessing obesity‐related dietary behaviours in young children

**DOI:** 10.1111/mcn.13613

**Published:** 2024-01-08

**Authors:** Lucinda Bell, Alexandra Manson, Dorota Zarnowiecki, Shi Ning Tan, Rebecca Byrne, Rachael Taylor, Miaobing Zheng, Li Ming Wen, Rebecca Golley

**Affiliations:** ^1^ Flinders University, College of Nursing and Health Sciences, Caring Futures Institute Adelaide South Australia Australia; ^2^ School of Exercise and Nutrition Sciences, Faculty of Health Queensland University of Technology Kelvin Grove Queensland Australia; ^3^ Department of Medicine University of Otago Dunedin New Zealand; ^4^ Institute for Physical Activity and Nutrition, School of Exercise and Nutrition Sciences Deakin University Geelong Victoria Australia; ^5^ Sydney School of Public Health, Faculty of Medicine and Health Sydney New South Wales Australia

**Keywords:** child, diet, dietary assessment, infant, obesity, obesity risk, reliability, validation

## Abstract

There are few short, validated tools to assess young children's obesity‐related dietary behaviours, limiting the rapid screening of dietary behaviours in research and practice‐based early obesity prevention. This study aimed to develop and assess the reliability and validity of a caregiver‐reported short dietary questionnaire to rapidly assess obesity‐related dietary behaviours in children aged 6 months to 5 years. The Early Prevention of Obesity in Childhood Dietary Questionnaire (EPOCH‐DQ) was developed using a rigorous process to determine content and structural validity. Three age‐appropriate versions were developed for (1) infants, aged 6–12 months, (2) toddlers, aged 1–2.9 years and (3) pre‐schoolers, aged 3–5 years. The questionnaire (7–15 items) measures dietary behaviours, including diet risk from non‐core food and beverage intake, diet quality from vegetable frequency, bread type and infant feeding practices. Test–retest reliability was assessed from repeated administrations 1 week apart (*n* = 126). Internal consistency, concurrent validity (against a comparison questionnaire, the InFANT Food Frequency Questionnaire), construct validity and interpretability were assessed (*n* = 209). Most scores were highly correlated and significantly associated (*p* < 0.05) for validity (*r*_s_: 0.45–0.89, percentage agreement 68%–100%) and reliability (intraclass correlation coefficient: 0.61–0.99) for diet risk, diet quality and feeding practice items. The EPOCH‐DQ shows acceptable validity and reliability for screening of obesity‐related behaviours of children under 5 years of age. The short length and, thus, low participant burden of the EPOCH‐DQ allows for potential applications in various settings. Future testing of the EPOCH‐DQ should evaluate culturally and socio‐economically diverse populations and establish the predictive validity and sensitivity to detect change.

## Key messages

- Accurate measurement of obesity‐related dietary behaviours is essential for the evaluation of early obesity prevention programmes and monitoring population trends.
- The 7–15 item Early Prevention of Obesity in Childhood Dietary Questionnaire (EPOCH‐DQ) was shown to have acceptable validity and reliability for assessing obesity‐related behaviours of children aged 6 months to 5 years of age.
- The short length and thus low participant burden of the EPOCH‐DQ allows for potential applications in various settings, including research, population health monitoring and use by health professionals.
- Future testing of the EPOCH‐DQ should include evaluation across culturally and socio‐economically diverse populations and determine the predictive validity and sensitivity to detect change of the EPOCH‐DQ.

## Graphical abstract

The 7–15 item EPOCH‐DQ was shown to have acceptable validity and reliability for assessing obesity‐related behaviours of children aged 6 months to 5 years of age. The short length and low participant burden allow for potential applications in various settings, including research, population health monitoring and use by health professionals.

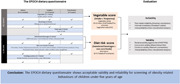

## 1 INTRODUCTION

Childhood obesity remains a significant health priority in Australia and globally (Department of Health, 2019; World Health Organization, 2016). Worldwide, an estimated 39 million children under 5 years of age are affected by overweight and obesity, as defined by body mass index (BMI) (kg/m^2^) (United Nations Children's Fund [UNICEF], World Health Organization, & World Bank Group, 2021; World Health Organization, 2021), highlighting the critical importance of the first 2000 days of life for obesity prevention. Overweight and obesity in childhood are associated with lower educational attainment, poorer quality of life, higher health care costs and chronic disease risk (Hayes et al., 2016). Obesity and associated behavioural risk factors established early in life may lay the foundation for poor lifelong habits, chronic disease risk and obesity trajectories (Llewellyn et al., 2016; Monasta et al., 2010). Research, policy and intervention efforts aimed towards preventing and reducing the development of childhood obesity are urgently needed (Harrison et al., 2011).

Dietary intake, alongside physical activity, sleep and screen time, are key modifiable behavioural risk factors for obesity in childhood (Mihrshahi & Baur, 2018). Dietary behaviours associated with an increased risk of obesity in childhood include excess intake of energy‐dense, nutrient‐poor foods and beverages (Ambrosini, 2014; Liberali et al., 2020; Poorolajal et al., 2020), defined here as non‐core foods. Poor diet quality, characterised by low vegetable intake and excess milk intake in infancy, may also contribute to an increased risk of obesity in early childhood (Appleton et al., 2018; Binns et al., 2007; Poorolajal et al., 2020). Excess non‐core food intake can displace core foods in the diet (National Health and Medical Research Council, 2013). In Australia, by 2–3 years of age, over 30% of a child's energy intake comes from non‐core foods such as sweet baked products, chocolate, salty snack foods and fried foods, and almost all (99.4%) children do not meet the recommendations for vegetable intake (Australian Institute of Health and Welfare, 2018). Concerningly similar patterns in young children's dietary intake are observed internationally (Keast et al., 2013; Public Health England, 2020).

Accurate measurement of obesity‐related dietary behaviours is essential for the evaluation of early obesity prevention programmes and monitoring population trends. There are many methods to assess children's dietary intake, with advantages and disadvantages for use across practice, policy and research settings (Krijger et al., 2022; Magarey et al., 2011). Dietary assessment methods such as diet histories, weighed food records and 24‐h recalls provide accurate and detailed information (Dao et al., 2019). However, these methods are time‐intensive, costly and burdensome for respondents, limiting their usefulness within policy and practice settings (Magarey et al., 2011). Food Frequency Questionnaires (FFQs) and short dietary questionnaires are self‐administered dietary assessment methods that are developed to measure specific aspects of diet. Short dietary questionnaires, typically 15 questions or fewer in length, provide an alternative to longer FFQs in settings where brief tools are needed (Burrows et al., 2012).

Short dietary questionnaires, or individually validated survey items, are an appealing approach to monitoring population dietary intake and evaluating intervention programmes in practice and at scale. Short questionnaires can be tailored to outcomes of interest, be administered quickly in a variety of formats and have low respondent burden. Further, they allow for easy comparison of food group intake against dietary guidelines (National Institutes of Health National Cancer Institute, n.d.; NIHR Cambridge Biomedical Research Centre, n.d.). Existing short questionnaires validated in Australian populations assess various aspects of diet, predominantly diet quality against dietary guidelines and have been developed for populations ranging from 12 months to 16 years of age (Bell et al., 2013; Dutch et al., 2023; Golley et al., 2017). For example, the 28‐item Children's Dietary Questionnaire assesses the intake of 4–16‐year‐old children for positive indicators (i.e., vegetables, fruit, water, reduced fat products) and negative indicators (i.e., high fat/high sugar foods and beverages) of diet quality (Magarey et al., 2009). The Toddler (ages 12–36 months) and Pre‐schooler (3–5 years) Dietary Questionnaires (19 items) assess the whole diet intake, including core and non‐core foods, against dietary guidelines and provide an assessment of dietary risk (Bell et al., 2014, 2018; Magarey et al., 2009). Similarly, the 10‐item Child Food and Beverage Intake Questionnaire, developed for American 2–4‐year‐old, assesses the intake of fruits, vegetables, sweetened foods and beverages (Koleilat & Whaley, 2016). However, none of these tools are designed for rapid screening of children's health behaviours across the early life period (birth – 5 years), limiting the capacity to identify risk and inform targeted intervention for obesity prevention in policy and practice settings.

Further to this, a recent review highlighted a lack of validated and reliable short diet questionnaires that measure obesity‐related dietary behaviours in young children (<5 years) (Byrne et al., 2019). Reviews of short dietary questionnaires highlight the lack of validated and ‘fit‐for‐purpose’ dietary assessment tools for use with infants, toddlers and pre‐school‐aged children that are feasible for use in policy and practice settings as a screening instrument to identify behaviours that place children at higher risk of poor growth, health and development (Byrne et al., 2019; Dutch et al., 2023; Golley et al., 2017). Therefore, this study aimed to (1) develop a short dietary questionnaire feasible for use in research and practice that assesses obesity‐related dietary behaviours of young children aged 6 months to 5 years and (2) examine the validity and reliability of the short dietary questionnaire.

## 2 METHODS

The first step of this study was the development of the Early Prevention of Obesity in Childhood Dietary Questionnaire (EPOCH‐DQ). The EPOCH‐DQ is an age‐appropriate, parent‐completed short dietary questionnaire that assesses obesity‐related dietary behaviours over the previous 7 days. Three age‐appropriate versions were developed, suitable for use with children aged 6–12 months (infant version), 1–2.9 years (toddler version) and 3–5 years (pre‐school version). Domains that collectively place an individual at a higher risk of obesity were captured, as it is overall diet quality that is most important to obesity risk (Okubo et al., 2015). For an instrument to be considered valid, it should be grounded in theory, precise and dependable, accurate within performance standards, and suited for the purpose and context (Frongillo et al., 2019). Accordingly, the EPOCH‐DQ was developed using a rigorous process (Kirkpatrick et al., 2019; Townsend, 2006) to determine content and structural validity before the extensive statistical analysis of concurrent validity, construct validity, internal consistency, interpretability and test–retest reliability were assessed.

### 2.1 Development of the EPOCH‐DQ

The development of the EPOCH‐DQ is summarised below and in Supporting Information: Figure S1.

#### 2.1.1 Content validity—Selection of domains and items

Content validity, the degree to which the content of an instrument reflects the construct which it intends to measure (Mokkink et al., 2010), was utilised for the development of the EPOCH‐DQ and involved the selection of the domains and items for inclusion in the questionnaire using a multi‐stage process. The first stage involved the collation of dietary guidelines (National Health and Medical Research Council, 2012, 2013), existing evidence and recommendations on obesity risk behaviours in early childhood (Ambrosini, 2014; Appleton et al., 2018; Matvienko‐Sikar et al., 2019). This, for example, allowed the identification of guideline recommendations for vegetable intake and variety in children 6 months to 5 years of age. The second stage involved a review and mapping of existing short questionnaires to determine whether any could be adapted for use and subsequently to identify candidate domains and items for inclusion in the EPOCH‐DQ. No existing short dietary questionnaires which exclusively measured aspects of dietary intake related to increased obesity risk were identified, as confirmed by a systematic review (Byrne et al., 2019). The potential items designed to assess food intake and food behaviours were mapped from the identified short questionnaires (Bell et al., 2014, 2018; Bennett et al., 2009; Hendrie et al., 2014; Magarey et al., 2009) and four key early feeding intervention studies (Campbell et al., 2008; Magarey et al., 2016; Taylor et al., 2011; Wen et al., 2012). These questions were incorporated with the collated evidence on diet risk behaviours. The third stage involved an iterative process of screening, discussion and exclusion of the evidence by reviewers from the EPOCH Centre for Research Excellence (Centre of Research Excellence In Early Childhood Prevention Obesity, 2021). Clinicians and researchers with expertise in early childhood obesity and dietary assessment methodology were consulted to refine and prioritise the item pool to ensure the brevity of the tool. This stage resulted in the selection of items representing elements of diet quality (vegetable intake, bread type, dairy type) and dietary risk (non‐core foods and beverages). The fourth stage gathered insights from clinicians within the EPOCH Centre for Research Excellence, including those working on scaling up obesity prevention interventions, who identified further items relating to feeding practices (bottle finished, feeding frequencies, quantity and duration) to add to the item pool. The resulting item pool informed the domains and items used in the EPOCH‐DQ (described below).

#### 2.1.2 Face validity—Pre‐testing of questions

Face validity, the degree to which an instrument appears to be an accurate reflection of the constructs intended to be measured (Mokkink et al., 2010), was determined by pre‐testing short food question styles used in existing tools to determine their suitability and acceptability. Pre‐testing involved cognitive interviewing conducted with 21 parents of young children, as reported by Zarnowiecki et al. (2020), to understand how parents recall information when reporting their children's food intake and to refine question‐wording to improve readability and acceptability, and therefore improve face validity of the EPOCH‐DQ (Zarnowiecki et al., 2020). Parents preferred a shorter reporting timeframe and tended to mostly consider the past week of intake, even when prompted to recall intake over a longer period of time (e.g., 1 month) (Zarnowiecki et al., 2020). The use of the term ‘usual’ intake presented a tension for parents between a desire to report ‘best’ usual intake versus actual usual intake. Accordingly, a 7‐day reporting period was selected for the EPOCH‐DQ, and the term ‘usual’ was omitted from questions. Of the three different styles of short food questions presented to parents in the interviews (Bell et al., 2014; Hendrie et al., 2014; Magarey et al., 2009), reporting intake frequency (as per the Children's Dietary Questionnaire [Magarey et al., 2009]) rather than portion size was most preferred by parents (Zarnowiecki et al., 2020). Therefore, this style of question was used in the EPOCH‐DQ.

### 2.2 Content of the EPOCH‐DQ

The content and face validity measures subsequently informed the tool content and question styles of the EPOCH‐DQ, developed to assess the intake of a subset of foods and beverages associated with an elevated risk of obesity. Intake is assessed by measuring the consumption frequency of nine non‐core foods and beverages (1. fruit juice, fruit drinks, soft drinks and cordial, 2. flavoured milk drinks, 3. chocolate, 4. potato crisps, savoury biscuits, 5. ice cream and ice blocks, 6. fried potato products, 7. pizza, 8. processed meat, 9. sweet biscuits, cakes, muffins, buns and doughnuts), hereby classified as the ‘diet risk’ items. In the 1–2.9 years (toddler) and 3–5 years (pre‐school) versions, parents report children's intake of diet risk items over the past 7 days on a 7‐point scale reflecting frequency of intake ranging from ‘did not have’ to ‘8+ times’. The 6–12‐month (infant) version asks parents to report intake over the past 7 days on the same food items using yes/no response options. The weekly frequency of the seven diet risk foods is summed to form the non‐core foods score (range, 0–7 infant, 0–56 toddler and pre‐school versions), and two diet risk beverage items are summed to form the sweetened beverages score (range, 0–2 infant, 0–16 toddler and pre‐school). All nine diet risk items are also summed to form the diet risk score (range, 0–9 infant, 0–72 Toddler and pre‐school), which considers the frequency of all diet risk items consumed over the week.

The items that reflect ‘diet quality’ include vegetable intake and variety, type of bread consumed and type of dairy consumed. Vegetable intake is reported as daily frequency over the past 7 days on a 7‐point scale ranging from ‘did not have’ to ‘6+ times’ (i.e., Vegetable frequency). Vegetable variety is reported as a tick‐box response of vegetables consumed over the past 7 days from a list of 19–26 items, depending on the version used (Supporting Information: Table S3). Bread type (1. Does not eat bread, 2. White bread, 3. High‐fibre white bread, 4. Wholegrain bread sometimes, 5. Wholegrain bread most of the time, 6. Wholegrain bread always), included in all versions, and dairy type (1. Does not drink milk, 2. Whole milk, 3. Low‐fat milk, 4. Skim milk, 5. Soy milk, 6. Other kinds of milk), included in the 1–2.9 years (toddler) and 3–5 years (pre‐school) versions only, are reported as six‐option multiple choice questions. Additional questions can be added to measure feeding practices associated with increased risk of obesity (Matvienko‐Sikar et al., 2019). These include a six‐item multiple choice response on the frequency of encouragement to finish a bottle, cow's milk and formula quantities and frequency and durations of formula and breastfeeding. These questions are included for clinical application, and risk can be evaluated in the context of whole diet and child growth. All ‘diet risk’ and ‘diet quality’ items are classified based on alignment with recommendations in the Australian Dietary Guidelines (ADGs) for infants, toddlers and children (National Health and Medical Research Council, 2012, 2013), identified weight gain‐related behaviours (Appleton et al., 2018), and compared to a core outcome set for childhood obesity prevention (Matvienko‐Sikar et al., 2019). These classifications are used to compare children's current dietary intake to recommendations to determine if the child is at dietary risk.

### 2.3 Reliability and validity of the EPOCH‐DQ

#### 2.3.1 Study design

Two online validation studies were conducted between October 2019 and July 2021 to determine the validity and reliability of the EPOCH‐DQ: (1) study 1 validating the infant version for 6–12 months olds and (2) study 2 validating the toddler and pre‐school versions for children aged 1–2.9 and 3–5 years, respectively (subsequently referred to as the 1–5‐year‐old versions). The same methods were used for both studies, except for minor differences, as noted below.

#### 2.3.2 Study participants

Participants were parents of infants aged 6–12 months, toddlers aged 1–2.9 years and pre‐schoolers aged 3–5 years, living in Australia. Parents were recruited via social media advertisements, sharing study information on relevant social media pages, distribution of flyers around the University, childcare centres and libraries, and direct email of study information to a database of parents interested in research participation. Children with multiple food allergies or restrictive dietary requirements due to medical conditions were excluded. Parents with more than one eligible child were instructed to choose one child to participate in the study. Parental consent was obtained, and those who completed both study time points were offered a chance to win an AUD$30 supermarket voucher in recognition of their time. A sample size requirement of 100 participants for each validation study (1 and 2) was set based on sample size recommendations for validation studies and for enabling Bland–Altman method analysis (Cade et al., 2002). This sample size also met the requirements for repeatability analysis using intraclass correlation coefficients, assuming test–retest reliability correlations of 0.7–0.9 as per previous research (Bell et al., 2014; Magarey et al., 2009; Zou, 2012).

#### 2.3.3 Data collection

Data was collected on two occasions, with all questionnaires administered online using Qualtrics CoreXM software (Qualtrics). At T1, parents completed brief questions via an email survey link to establish study eligibility, a demographic questionnaire, the EPOCH‐DQ (i.e., the relevant age‐appropriate version), and the validated InFANT FFQ (Zheng et al., 2020) (details outlined below). To examine reliability, parents completed the EPOCH‐DQ for a second time (T2) 7 days after T1 completion. Two email reminders were sent to participants who did not complete T2 (the last reminder on Day 12).

##### InFANT FFQ

To examine the validity of the EPOCH‐DQ, an FFQ was chosen as the comparative dietary assessment tool for measuring concurrent and construct validity, given they have the same error structure and both can measure usual intake (Collins et al., 2010; Magarey et al., 2011). The 68‐item InFANT FFQ measures usual total dietary intake, including non‐core foods and beverages, and has been validated against three 24 h recalls in Australian children aged 1.5, 3.5 and 5.0 years, showing fair agreement with nutrient and food intake (Zheng et al., 2020). The InFANT FFQ takes approximately 20 min to complete, with response options ranging from monthly to daily intake frequencies. Responses from the InFANT FFQ ranging from ‘never or less than once a month’ to ‘6 or more times a day’ were combined and recoded to determine weekly frequency, equivalent to the EPOCH‐DQ. The InFANT FFQ was also used as the reference instrument for the infant version of the EPOCH‐DQ as no suitable FFQ was identified that was both validated with Australian infants and included all non‐core foods and beverages of interest. A question was added from the validated POI FFQ (Watson et al., 2015) about the intake of commercially prepared baby foods to assess vegetable intake from this source.

##### Demographic questionnaire

Child and parent demographic characteristics were collected at T1 to understand the study sample. Child characteristics included sex, age, and parent‐reported length/height and weight. Child BMI was calculated from height and weight and converted to age and sex‐adjusted BMI *z*‐scores by the least mean squares method using World Health Organization (2006) growth standards in the WHO (2011) Anthro Software Version 3.2.2 add‐in. Parent characteristics included educational level, household income, marital status, employment status and postcode (see Table 1). Postcodes were classified using the Socioeconomic Indexes for Areas (SEIFA) 2016, Index of Relative Socio‐economic Advantage and Disadvantage, as an indicator of area‐level socioeconomic status (Australian Bureau of Statistics, 2016).

**Table 1:** Characteristics of the sample of 6–60‐month‐olds who completed the EPOCH‐DQ.

	Study 1 (*n* = 113)				Study 2 (*n* = 96)			
	T1		T2		T1		T2	
	*n*	%	*n*	%	*n*	%	*n*	%
Carer characteristics								
Age (years)^a^	32.0	4.3	31.6	4.1	34.0	5.0	34.7	4.16
Sex – Female	112	99.1	70	100.0	91	94.8	50	96.2
Child characteristics								
Age (months)^a^	8.7	1.9	8.7	2.0	35.8	13.1	34.3	13.1
Sex – Female	54	47.8	33	47.1	46	47.9	23	44.2
Weight category^b^								
Underweight	14	12.8	10	14.3	7	8.0	3.0	5.8
Normal weight	64	58.7	43	61.4	41	47.1	25.0	48.1
Overweight	11	10.1	8	11.4	14	16.1	10.0	19.2
Obesity	20	18.3	9	12.9	25	28.7	14.0	26.9
No. of children at home								
One	79	69.9	57	81.7	37	38.5	25	48.1
Two	25	22.1	9	12.9	39	40.6	18	34.6
Three or more	9	8.0	4	4.7	20	20.8	9	17.3
Uses external child care services	28	24.8	17	24.3	79	85.9	43	78.2
Marital status								
Single parent	5	4.4	3	4.3	6	6.5	1	1.9
Co‐parent	108	95.6	67	95.7	86	93.5	51	98.1
Carer education level								
Did not complete high school	1	0.9	1	1.4	3	3.1	1	1.9
Completed high school	8	7.1	4	5.7	9	9.4	6	11.5
Tech/trade qualification	21	18.6	11	15.7	15	15.6	7	13.5
Tertiary degree or higher	83	73.5	54	77.1	65	66.7	38	73
AUS state of residence								
ACT	4	3.5	3	4.3	3	3.1	2	3.8
NSW	30	26.5	20	28.6	12	12.5	9	17.3
NT	2	1.8	2	2.9	1	1.0	1	1.9
QLD	11	9.7	6	8.6	7	7.3	1	1.9
SA	37	32.7	25	35.7	48	50.0	27	51.9
TAS	‐	‐	‐	‐	5	5.2	1	1.9
VIC	21	18.6	7	10.0	18	18.8	11	21.2
WA	8	7.1	7	10.0	2	2.1	‐	‐
Employment status								
Full time employment	41	36.3	27	28.6	21	22.8	12	23.1
Part time employment	32	28.3	19	27.1	50	54.3	31	59.6
Home maker	30	26.5	19	27.1	18	18.8	7	13.5
Student	6	5.3	3	4.3	2	2.1	2	3.8
Unemployed	4	3.6	2	2.8	1	1.1	‐	‐
Household income range^c^								
AUD < 52,000	6	5.3	4	5.7	8	8.8	3	5.7
AUD 52,000–77,999	12	10.6	9	12.9	9	9.8	4	7.7
AUD 78,000–103,999	21	18.6	13	18.6	16	17.4	8	15.4
AUD 104,000–114,399	11	9.7	7	10.0	8	8.7	7	13.5
>AUD 114,400	55	48.7	32	45.7	42	45.7	25	48.1
SEIFA								
Deciles 1, 2	12	10.6	8	11.4	13	14.1	6	11.5
Deciles 3, 4	26	23.0	15	21.4	17	18.5	8	15.4
Deciles 5, 6	22	19.5	16	22.9	18	19.6	11	21.2
Deciles 7, 8	26	23.0	15	21.4	27	29.3	17	32.7
Deciles 9, 10	27	23.9	16	22.9	17	18.5	10	19.2
Time between T1 and T2 (days)^a^	8.4	3.6			9.3	2.8		

*Note*: Study 1 used the EPOCH‐DQ 6–12 months version. Study 2 used the EPOCH‐DQ 1–5‐year‐old version. T1 and T2 refers to Timepoint 1 and 2 of questionnaire repetitions. AUD, Australian dollar, Australian Median total income was $52,338 in 2019/2020 (Australian Bureau of Statistics, 2015–16 to 2019–20).

Abbreviations: ACT, Australian Capital Territory; AUD, Australian dollar; AUS, Australia; EPOCH‐DQ, Early Prevention of Obesity in Childhood Dietary Questionnaire; NSW, New South Wales; NT, Northern Territory; QLD, Queensland; SA, South Australia; SEIFA, Index of Relative Socio‐economic Advantage and Disadvantage; TAS, Tasmania; VIC, Victoria; WA, Western Australia.

^a^ These data are presented as mean and standard deviation instead of the number of participants and percentage of the sample.

^b^ Missing, *n* = 3 at Timepoint 1 study 1, Participants had biologically implausible values according to the WHO Percentiles. Weight category determined using parent‐reported data.

^c^ Did not report *n* = 8 at at Timepoint 1 study 2, *n* = 9 Timepoint 1 study 1.

### 2.4 Statistical analysis

All analyses were conducted using IBM SPSS Statistics Version 25. To assess reliability and validity, correlations were interpreted as high (≥0.7), moderate (0.5–0.69) and low (<0.5), as suggested by Hinkle et al. (1994). These interpretations are consistent with diet validation studies in similar populations (Bell et al., 2014). The level of significance was set at *p* < 0.05. Scale variables were assessed for normality by comparing the mean and median values, analysis of skewness and kurtosis *z*‐scores, and visual assessment of the histogram and plotted normal curve.

#### 2.4.1 Validity

##### Concurrent validity of the EPOCH‐DQ against a comparison FFQ

Concurrent validity is the degree to which the scores of an instrument are an adequate reflection of an established measure (Boateng et al., 2018). A Bland–Altman plot was constructed to assess the strength of agreement between the EPOCH‐DQ and the InFANT FFQ by plotting the mean difference between the questionnaires against the mean score for the diet risk score. The plot was visually assessed, and linear regression analysis was performed to test for systematic bias. Data points were used to determine the mean bias and the limits of agreement (±2 standard deviation [SD]) of the mean bias (Lombard et al., 2015).

##### Construct validity of the EPOCH‐DQ

Construct validity is the degree to which the scores are consistent with hypotheses based on the assumption that the instrument measures desired constructs in a valid manner (Mokkink et al., 2010). Data from the 2007 National Children's Nutrition and Physical Activity Survey (NCNPAS) were used to assess nutrient composition and median portion size of FFQ items (South Australia Department of Health, 2007). Pearson's correlation was used to explore associations between the reported intake frequency for scale items and vegetable intake and the calculated nutrient intakes. Nutrient intake data were normally distributed and met the assumptions of Pearson's correlation coefficient for distribution and linearity. Construct validity of the EPOCH‐DQ infant version was not evaluated as nutrient intake data for infants was not available in the NCNPAS (South Australia Department of Health, 2007).

To assess validity at the item and scale levels against the InFANT FFQ, Spearman's correlations were used. The validity of diet risk items for the EPOCH‐DQ infant version was assessed through percentage agreement as data did not meet alternative test assumptions due to the low frequency of positive values and limited data spread (Pallant, 2003). Validity of diet quality measures, including vegetable frequency, vegetable variety, vegetable variety classification, and vegetable score (i.e., frequency + variety), were assessed at the item level for all EPOCH‐DQ versions. Variety was determined through the number of different vegetables consumed across the week (e.g., carrot + cucumber = 2; avocado + mushroom + broccoli = 3). Vegetable variety classification was calculated by classifying vegetables consumed into one of the ADG vegetable categories (i.e., dark green, orange, salad, starchy, legumes, other) and summing the number of categories consumed (score range, 0–6) (Supporting Information: Table S2) (National Health and Medical Research Council, 2012, 2013).

The *χ* ^2^ independence test was used to assess validity at the item level for diet quality categorical items (e.g., bread and dairy type responses) against the InFANT FFQ (McHugh, 2013). Although the data violated the assumptions of cell frequency (Pallant, 2003), these results are reported due to a lack of suitable alternatives and to allow for comparison to the previous literature. However, results should be interpreted with discretion.

#### 2.4.2 Reliability

##### Test–retest reliability of EPOCH‐DQ administrations

Test–retest reliability is the extent to which scores are consistent across repeated test administrations completed on different occasions by the same participants (Mokkink et al., 2010). Test–retest reliability between administrations of the EPOCH‐DQ diet risk, diet quality and feeding practices items were assessed using intraclass correlation coefficient (ICC) linear mixed model with the absolute agreement, two‐way mixed effects (Koo & Li, 2016). The reliability of categorical items (dairy type, bread type and encouragement to finish bottle) was assessed using Cohen's kappa (*κ*) and percentage agreement, where *κ* ≥ 0.8 was considered good reliability (McHugh, 2012). Percentage agreement of vegetable items was used to determine the agreement between the EPOCH‐DQ administrations.

##### Internal consistency of dietary risk score

Internal consistency is the degree of interrelatedness among items, determining how well scale items fit together (Tavakol & Dennick, 2011). Cronbach's *α* was calculated for diet risk (9–10 items) and vegetable variety (19–26 items) scores. Generally, *α* > 0.7 are considered acceptable (Gleason et al., 2010), although if the number of scale items is less than 10, lower values are considered appropriate (Bland & Altman, 1997; Pallant, 2003).

##### Interpretability of EPOCH‐DQ scoring

Interpretability is the degree to which one can assign connotations to a measure's quantitative score (Mokkink et al., 2010). Cross‐classification was used to determine the percentage of participants consistently classified into the same risk category between EPOCH‐DQ T1 and T2 administrations and between the EPOCH‐DQ T1 administration and the InFANT FFQ.

### 2.5 Ethical statement

Ethical approval was granted by the Flinders University Social and Behavioural Research Ethics Committee, Adelaide, Australia (SBREC 8381).

## 3 RESULTS

### 3.1 Participant characteristics

**Figure 1:**
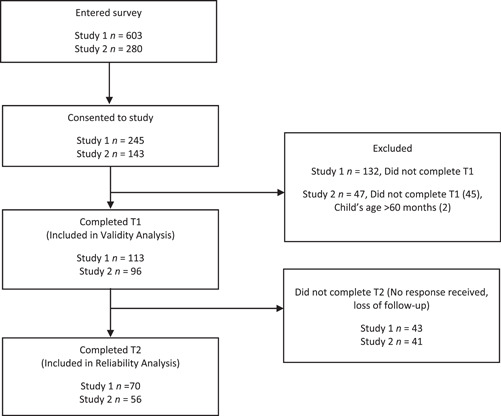
Flow chart of participants in the validation and reliability study of the Early Prevention of Obesity in Childhood Dietary Questionnaire (EPOCH‐DQ). Study 1 used EPOCH‐DQ 6–12 months version. Study 2 used EPOCH‐DQ 1–5‐year‐old versions.

Of the 603 (6–12 months) and 280 (1–5 years) participants who commenced the surveys, 113 (40.3% response rate) and 96 (15.9% response rate) completed T1 for study 1 and study 2, respectively (Figure 1). Of these, 70 (61.9%) and 56 (58.3%) participants, respectively, completed the survey at T2. Table 1 presents the sample characteristics. T1 and T2 questionnaires were completed a mean of 8.4 (SD, 3.6) days apart, (study 1) ranging from 6.8 to 30.9 days and 9.3 (SD, 2.8) days (study 2), ranging from 5.3 to 16.1 days. Participant characteristics for both questionnaires were similar between time points; however, parents with fewer children were more likely to complete T2 questionnaires. Participants for study 1 and study 2 were from all states in Australia, excluding Tasmania for study 1.

### 3.2 Validity

The Bland–Altman plots (Supporting Information: Figures S2 and S3) showed that >90% of participants lay within the limits of agreement (−2.49–2.45, study 1, and −15.23–7.82, study 2). The mean difference was −0.02 and −3.70 for the infant and 1–5‐year‐old versions, respectively, with a negative bias. There was a significant difference (*p* = 0.001) and negative linear bias for the 1–5‐year‐old versions, indicating that at higher serves, this version underestimated diet risk item intake by −4.5 serves/week (equivalent to <1 serves/day). For participants who consumed <14 servings of diet risk items per week, the mean bias was reduced to −2.5 serves/week. There was no significant linear trend for the fitted regression line for the infant version.

Table 2 shows the results for construct validity of the 1–5‐year‐old versions (study 2). Pearson's correlations showed diet risk scores, including non‐core foods and sweetened beverages, were significantly positively correlated with total energy, saturated fat, total sugars, dietary fibre, sodium and potassium (Table 2). Vegetable frequency was not significantly correlated with any nutrients but was correlated with dietary fibre intake.

**Table 2:** Construct validity of the EPOCH‐DQ 1–5‐year‐old versions subscales and discretionary subgroup against the calculated nutrient intakes from the InFANT FFQ data (*n* = 96).

Pearson's correlation coefficient							
Subscales	Total energy	SFA	Total sugars	Dietary fibre	Sodium	Potassium	Calcium
Sweetened beverage	**0.508** **	**0.368** *	**0.530** *	**0.339** **	**0.424** **	**0.403** **	0.160
Non‐core foods	**0.495** **	**0.350** *	**0.357** *	**0.309** **	**0.501** **	**0.231** *	0.160
Diet risk score	**0.575** **	**0.410** *	**0.472** *	**0.366** **	**0.551** **	**0.326** *	0.074
Vegetable frequency	−0.072	−0.188	0.103	**0.329** *	−0.110	0.116	−0.127

*Note*: Interpretation: ≥0.7, high correlation; 0.51–0.69, moderate correlation; ≤0.5, low correlation. Bold text indicates significant values.

Abbreviations: EPOCH‐DQ, Early Prevention of Obesity in Childhood Dietary Questionnaire; FFQ, Food Frequency Questionnaire.

* *p* < 0.05 (two‐tailed);

** *p* < 0.001 (two‐tailed).

Tables 3 and 4 show results for the validity of studies 1 and 2, respectively, against the InFANT FFQ. All Spearman's *ρ* correlations ranged from 0.47 to 0.69 and were significant (Tables 3 and 4). Most items had a moderate or high correlation (>0.51). The percentage agreement for individual diet items (study 1) ranged from 68% to 100%, with eight items having >80% agreement. The cow's milk feeding practices items included in study 1 were highly correlated (*r* = 0.71), as were the formula frequency items (0.79). Bread and dairy‐type items were found to relate to the InFANT FFQ equivalent items with a *χ* ^2^ likelihood ratio of *p* < 0.001 (Table 3, study 1 and Table 4, study 2).

**Table 3:** Test–retest reliability and concurrent validity of study 1, comparing EPOCH‐DQ (infant version) and InFANT FFQ.

	Validity^a^			Reliability^a^			
EPOCH‐IDQ food items	InFANT (*n* = 113)	EPOCH‐DQ T1 (*n* = 113)	% of agreement	EPOCH‐DQ (*n* = 70)^b^		*κ*	% of agreement
Diet risk items							
Fruit Juice	5	8	95.6	3	3	0.65	97.1
Fruit drinks, soft drinks, cordial				0	0	0	100.0
Flavoured milk drinks	1	1	100.0	0	0	0	100.0
Chocolate	3	3	96.5	3	3	0.3	94.3
Potato crisps, savoury biscuits	40	22	68.1	14	17	0.63	87.1
Ice cream, ice blocks	10	4	87.6	2	0	0	97.1
Fried potato products	18	24	82.3	15	12	0.59	87.1
Pizza	3	8	93.8	3	3	−0.04	91.4
Processed meat	11	15	89.4	12	13	0.66	90.0
Sweet biscuits, cakes, muffins, buns, doughnuts	15	19	89.4	10	10	0.53	88.6
Bread type							
Does not eat bread	31	30	183.89^c^	22	18	0.631	71.4
White bread	7	10		4	6		
High‐fibre white bread	13	3		3	4		
Wholegrain bread sometimes	3	33		21	23		
Wholegrain bread most of the time	10	15		9	8		
Wholegrain bread always	49	22		11	11		
Bottle finished							
Always	‐	6	‐	3	0	0.585	68.1
Most of the time	‐	13		7	13		
Sometimes	‐	12		5	7		
Rarely	‐	14		10	8		
Never	‐	29		19	21		
Does not drink from a bottle	‐	39		25	20		
Diet risk (median, IQR)							95% CI
Sweetened beverages	0.0 (0.0, 0.0)	0.0 (0.0, 0.0)	‐	0.0 (0.0, 0.0)	0.0 (0.0, 0.0)	0.79^e^	0.66, 0.87
Non‐core foods	0.0 (0.0, 1.0)	0.0 (0.0, 2.0)	‐	0.0 (0.0, 2.0)	0.0 (0.0, 2.0)	0.90^e^	0.85, 0.94
*Diet risk score* c	1.0 (0.0, 1.0)	0.0 (0.0, 2.0)	0.45^d^	0.0 (0.0, 2.0)	0.0 (0.0, 2.0)	0.91^e^	0.85, 0.94
Diet quality (median, IQR)							
Vegetable frequency (times/day)	2.5 (2.5, 2.5)	2.5 (2.5, 2.5)	0.46^d^	2.0 (2.0, 2.0)	2.0 (2.0, 3.0)	0.38^e^, ^f^	0.01, 0.62
Vegetable variety	7.0 (5.0, 9.0)	11.0 (8.0, 13.0)	0.65^d^	11.0 (8.0, 13.0)	10.0 (7.5, 13.0)	0.87^e^	0.62, 0.77
Vegetable variety classification	5.0 (4.0, 6.0)	5.0 (5.0, 6.0)	0.50^d^	5.0 (5.0, 6.0)	5.0 (4.0, 6.0)	0.84^e^	0.74, 0.90
*Vegetable score (frequency + variety)*	7.0 (6.5, 8.5)	7.5 (7.0, 8.5)	0.57^d^	7.0 (6.0, 8.0)	7.0 (6.0, 8.0)	0.70^e^	0.54, 0.81
Feeding practices (median, IQR)^h^							
Breastfeeding duration (weeks)	‐	30.1 (25.6, 38.8)	‐	26.7 (16.0, 36.2)	31.1 (26.9, 40.3)	>0.99^e^	0.97, 0.99
Age of first formula feed (weeks)	‐	1.0 (0.0, 8.0)	‐	1.0 (0.0, 11.0)	1.0 (1.0, 7.5)	0.98^e^	0.96, 0.99
Quantity of formula consumed each time (mLs)	‐	160.0 (150.0, 195.0)	‐	170.0 (150.0, 197.5)	150 (60.0, 200.0)	0.91^e^	0.80, 0.96
Formula frequency (times/week)	17.5 (3.0, 31.5)	16.0 (3.0, 32.0)	0.79^d^	8.0 (2.5, 32.0)	7.0 (0.5, 24.0)	0.82^e^	0.63, 0.91
Formula quantity/week (frequency × mL)	‐	2400.0 (500.0, 5760.0)	‐	1400.0 (550.0, 5280.0)	840.0 (90.0, 4320.0)	0.87^e^	0.73, 0.94
Age of first cows milk introduction (weeks)	‐	30 (23.0, 44.0)	‐	32.0 (7.5, 312.5)	32.0 (24.0, 48.0)	0.99^e^	0.94, 0.99
Quantity of cows milk (mLs)	‐	40.0 (22.0, 87.5)	‐	50.0 (7.5, 75.0)	15.0 (60.0, 200.0)	0.83^e^	0.20, 0.97
Cows milk frequency (times/week)	3.0 (0.0, 7.0)	2.5 (0.0, 6.5)	0.39^d^, ^g^	2.0 (0.0, 7.0)	1.0 (0.0, 6.3)	0.92^e^	0.69, 0.98
Cows milk quantity/week (frequency × mL)	‐	30.0 (0.0, 270.0)	‐	60.0 (0.0, 875.0)	30.0 (0.0, 315.0)	0.88^e^	0.57, 0.97

*Note*: All values significant at *p* < 0.001 (2‐tailed), unless otherwise specified.

Abbreviations: CI, confidence interval; EPOCH‐DQ, Early Prevention of Obesity in Childhood Dietary Questionnaire; FFQ, Food Frequency Questionnaire; IQR, interquartile range.

^a^ Data presented as frequency of children consuming (*n*) or median (IQR).

^b^ Subgroup of participants completing both time points.

^c^ *χ* ^2^ likelihood ratios.

^d^ Spearman's *ρ* correlation coefficient (*r*_s_).

^e^ Intraclass correlation coefficient.

95% CI.

^f^ Significant at *p* < 0.05 (two‐tailed).

^g^ Not significant.

^h^ Number of children having formula *n* = 59, cows milk *n* = 22.

**Table 4:** Concurrent validity and test–retest reliability of Study 2, comparing EPOCH‐DQ (1–5‐year‐old versions) and InFANT FFQ.

	Validity^a^					Reliability^a^					
	InFANT (*n* = 96)		EPOCH‐DQ T1 (*n* = 96)			EPOCH‐DQ (*n* = 55)					
Diet risk items (times/week)	Median	IQR	Median	IQR	*r*_s_	T1 median	T1 IQR	T2 median	T2 IQR	ICC	95% CI
Sweetened beverages	0.9	0.0–3.5	1.0	0.0–3.0	0.89	1.0	0.0–2.0	1.0	0.0–2.0	0.97	0.94, 0.98
Fruit juice, fruit drinks, soft drinks, cordial	0.5	0.0–3.0	0.0	0.0–2.0	0.86	0.0	0.0–2.0	0.0	0.0–2.0	0.96	0.94, 0.98
Flavoured milk	0.0	0.0–0.5	0.0	0.0–1.0	0.67	0.0	0.0–0.8	0.0	0.0–1.0	0.92	0.86, 0.95
Non‐core foods	14.4	9.9–22.3	9.0	6.0–13.0	0.66	11.0	6.3–13.8	10.0	7.0–13.3	0.88	0.80, 0.93
Chocolate	0.5	0.0–1.0	1.0	0.0–2.0	0.66	1.0	0.0–2.0	1.0	0.0–2.0	0.87	0.77, 0.93
Potato crisp, savoury biscuits	5.0	3.0–7.9	3.0	1.0–4.0	0.50	3.0	2.0–4.0	3.0	2.0–4.0	0.82	0.69, 0.90
Ice cream, ice blocks	0.5	0.0–1.0	0.0	0.0–1.0	0.56	0.0	0.0–1.0	0.0	0.0–1.0	0.84	0.73, 0.91
Fried potato products	0.5	0.5–1.0	1.0	0.0–2.0	0.69	1.0	0.0–2.0	11.0	0.0–1.3	0.79	0.63, 0.88
Pizza	0.5	0.0–0.5	0.0	0.0–1.0	0.47	0.0	0.0–0.0	0.0	0.0–1.0	0.61	0.33, 0.77
Processed meat	1.5	0.9–3.5	2.0	1.0–3.0	0.73	2.0	1.0–3.0	2.0	0.5–2.5	0.87	0.77, 0.93
Sweet biscuit, cake, muffin, bun, doughnut	2.0	0.9–4.0	2.0	1.0–3.0	0.63	2.0	1.0–3.0	2.0	1.0–3.0	0.83	0.70, 0.90
Diet risk score	16.4	10.6–25.4	11.0	*7.0–16.0*	0.76	11.5	7.0–17.0	11.0	7.0–15.5	0.92	0.87, 0.96
Diet quality											
Vegetable frequency (times/day)	1.0	1.0–2.5	2.0	2.0–3.0	0.49	2.0	1.3–3.0	2.0	1.0–3.0	0.63	0.37, 0.79
Vegetable variety	8.0	6.0–10.0	11.0	8.0–14.0	0.62	12.0	9.0–16.0	10.0	8.0–13.0	0.89	0.73, 0.95
Vegetable variety classification	5.0	4.3–6.0	6.0	5.0–6.0	0.58	6.0	5.0–6.0	6.0	5.0–6.0	0.81	0.68, 0.89
*Vegetable score (frequency + variety)*	*7.0*	*6.0–7.5*	*8.5*	*6.5–8.5*	*0.60*	*8.0*	*6.0–8.75*	*8.0*	*6.0–8.0*	*0.80*	*0.65, 0.88*
Feeding practices^e^											
Breastfeeding duration (weeks)	‐	‐	54.0	36.0, 81.0		52.0	36.0, 80.0	56.0	40.0, 88.0	>0.99	0.99, >0.99
Age of first formula feed (weeks)	‐	‐	12.0	1.0, 29.0		16.0	1.0, 32.0	16.0	1.0, 34.0	>0.99	0.99, >0.99
Age of first cow milk introduction (weeks)	‐	‐	48.0	44.0, 52.0		48.0	46.0, 49.0	48.0	44.0, 52.0	0.98	0.95, 0.99
Quantity of cows milk (mLs)	‐	‐	150.0	100.0, 245.0		180.0	122.5, 250.0	125.0	100.0, 250.0	0.99	0.98, >0.99
Cows milk frequency (times/week)	7.0	3.0, 7.0	7.0	3.0, 8.0	0.71	6.0	2.0, 7.0	7.0	3.8, 7.3	0.89	0.75, 0.95
Cows milk quantity/week (frequency × mL)	‐	‐	875.0	350.0, 1470.0		930.0	312.5, 1485.0	837.5	550.0, 1575.0	0.96	0.91, 0.98
Dairy type (*n*, %)											
Does not drink milk	10	10.4	8	8.3	130.64^b^	5	9.1	2	2.1		0.61, 0.95
Whole milk	70	72.9	68	70.8		40	72.7	43	44.8		
Low Fat milk	5	5.2	6	6.3		3	5.5	5	5.2		
Skim milk	1	1.0	1	1.0		1	1.8	1	1.0	0.78^c^	
Soy milk	‐	‐	‐	‐		‐	‐	‐	‐	(90.9%)^d^	
Other milks	10	10.4	13	13.5		6	10.9	4	4.2		
Bread type (*n*, %)											
Does not eat bread	2	2.1	‐	‐	106.26^b^	‐	‐	‐	‐		0.54, 0.83
White bread	9	9.4	12	12.5		5	9.1	6	10.9		
High‐fibre white bread	11	11.5	9	9.4		6	10.9	5	9.1	0.69^c^	
Wholegrain bread sometimes	4	4.2	17	17.7		11	20.0	10	18.2	(76.4%)^d^	
Wholegrain bread most of the time	17	17.7	29	30.2		21	38.2	20	36.4		
Wholegrain bread always	53	55.2	29	30.2		12	12.8	14	25.5		

*Note*: Spearman's *ρ* correlation coefficient (*r*_s_) values and *χ* ^2^ likelihood ratios were significant at *p* < 0.01 (two‐tailed). Intraclass correlation coefficient and Cohen's *κ* values significant at *p* < 0.001 (two‐tailed).

Abbreviations: CI, confidence interval; EPOCH‐DQ, Early Prevention of Obesity in Childhood Dietary Questionnaire; FFQ, Food Frequency Questionnaire; ICC, intraclass correlation coefficient; IQR, interquartile range.

^a^ Data presented as or median, IQR or frequency of children consuming, %.

^b^ *χ* ^2^ likelihood ratio.

^c^ Cohen's *κ*.

^d^ Percentage agreement.

^e^ Participants of EPOCH‐DQ 1–2.9 years version, validity *n* = 49, reliability *n* = 33.

### 3.3 Test–retest reliability

Reliability indicators, including ICC, Cohen's *κ* and percentage agreement, are summarised in Table 3 for study 1 and Table 4 for study 2. All diet quality scales (*n* = 7, Table 3) for study 1 showed moderate to strong ICC (0.70–0.91), except for vegetable frequency, which showed a low but significant correlation (0.38). All feeding practice items had strong ICC (≥0.82) (study 1).

The ICC for non‐core foods and sweetened beverages for study 2 (*n* = 9, Table 4) ranged from 0.61 (pizza) to 0.97 (sweetened beverages). The ICC for diet quality items, including four vegetable scales, was moderate to strong (0.63–0.89) (study 2). For the individual vegetable items, there was a 66%–96% agreement between T1 and T2 (Supporting Information: Table S3, study 1 and study 2).

### 3.4 Internal consistency

Cronbach's *α* for the T1 vegetable items (*n* = 26, i.e., carrot, cucumber, lettuce) was between 0.715 and 0.798 for study 1 and study 2, respectively, and between 0.600 and 0.685 for diet risk items (*n* = 9) (data not presented).

### 3.5 Interpretability

As shown in Supporting Information: Table S1, classification analysis between the EPOCH‐DQ against the InFANT FFQ revealed that 62%–96% of participants were classified into the same risk categories, with a mean of 82% agreement. Between EPOCH‐DQ timepoints, 69%–97% of participants were consistently categorised with a mean of 84% agreement.

## 4 DISCUSSION

This paper describes the development and testing of a short dietary questionnaire measuring obesity‐related dietary behaviours in young children aged 6 months to 5 years. The EPOCH‐DQ tool is applicable as a screening tool for identifying dietary risk behaviours for use in settings where rapid assessment tools are required, such as population monitoring, scale up of community interventions and screening in primary health care settings. The dietary behaviours assessed were moderately to highly correlated and significantly associated between repeated administrations or when compared to a validated comparison tool, the InFANT FFQ. The EPOCH‐DQ can be considered as being a valid and reliable tool for screening obesity‐related dietary behaviours in young children aged 6 months to 5 years to inform targeted interventions.

A multi‐stage development and testing process was used to examine the validity of the EPOCH‐DQ. Content validity was considered through a combination of a systematic literature review, mapping of existing tools and expert insight, and consensus to identify prioritised items for inclusion to ensure tool brevity. Cognitive interviewing with parents of young children provided insights for preferred question styles, wording of questions to improve understanding and prompts to increase reporting accuracy (Zarnowiecki et al., 2020). Construct validity was established for the 1–5‐year‐old versions of the EPOCH‐DQ, showing moderate correlations with nutrient intakes in expected directions (National Health and Medical Research Council, 2013). As the EPOCH‐DQ did not measure total diet, correlations of moderate magnitude provide an indication of good construct validity.

The EPOCH‐DQ performed well in terms of relative validity, meeting or exceeding the results of previous validation studies. The total scale items derived from the EPOCH‐DQ and the reference FFQ were highly correlated. Individual items also showed moderate to high correlations and no significant differences for most items. Validation of existing short questionnaires in young children has demonstrated Spearman's correlations in the magnitude of 0.33–0.74 for vegetable intake, 0.31–0.66 for non‐core foods and 0.55–0.88 for sugar‐sweetened beverages and fruit juice (Bennett et al., 2009; Huybrechts et al., 2009; Magarey et al., 2009). The Bland–Altman plots, when interpreted in the context of the tool and its scoring, revealed narrow limits of agreement and limited mean bias, indicating that the dietary questionnaire can appropriately identify diet risk item intake at the individual level. There was no systematic bias for the infant version, with the 1–5‐year‐old versions underestimating intake of dietary risk items by approximately half a serve per day when reporting higher frequencies compared with the reference FFQ. The mean bias was notably lower for frequencies of <14 times per week, meaning the EPOCH‐DQ accurately reflects the level of specificity required for dietary guideline compliance assessment. Additionally, there was a significant and high correlation for Spearman's *ρ*, indicating the presence of a strong relationship between the diet risk score EPOCH‐DQ and InFANT FFQ responses.

The poorest performing item across all EPOCH‐DQ versions was vegetable frequency, consistent with other validation studies that have found poor validity for vegetable items (Huybrechts et al., 2009; Koleilat & Whaley, 2016; Magarey et al., 2009). Possible reasons for poorer reporting of vegetable intake may include high variability in child intake and food preferences in early childhood, with a considerable proportion of young children experiencing food neophobia, as well as misreporting of vegetables that are consumed by children at childcare or in mixed dishes (e.g., ‘hiding’ vegetables) (Mauch et al., 2017; Wallace et al., 2018). Insights from cognitive interviewing suggest that parents find it harder to recall foods that are regularly consumed, that is, core foods such as grains and vegetables, and easier to recall foods consumed less frequently or on special occasions, such as non‐core foods (Zarnowiecki et al., 2020). Therefore, alternative strategies should be considered to improve reporting of core foods such as vegetables. In this study, better validity was observed for reporting types of vegetables consumed from a list versus reporting vegetable frequency, suggesting that it may be easier for parents to recall types of vegetables in comparison to the amount consumed. This is consistent with the recall strategies used by parents in the cognitive interviews, such as recalling food shopping, contents of the refrigerator, or foods prepared at mealtimes (Zarnowiecki et al., 2020). Therefore, the inclusion of vegetable variety questions, in place of or in combination with vegetable frequency, may provide a better estimate of intake and identify a practical strategy to increase vegetable intake (e.g., increasing vegetable variety).

Overall, the EPOCH‐DQ performed well in terms of test–retest reliability. Intraclass correlations ranged from 0.61 for pizza to 0.99 for breastfeeding duration, which was equal to or exceeded test–retest reliability estimates for existing short dietary questionnaires completed by parents reporting young children's intake. For example, test–retest reliability ranges from 0.51 to 0.90 for ICC in diet questionnaire validation studies in children of similar age (Bell et al., 2014; Magarey et al., 2009). The percentage agreement for the individual items was considered appropriate compared to previous similar short dietary questionnaire validation studies (Bell et al., 2014; Flood et al., 2014). The vegetable items for the EPOCH‐DQ ranged from 66% to 100% agreement (*n* = 10–26 items), compared to 32%–86% for *n* = 19 items (Bell et al., 2014), and 53%–97%; *n* = 16 (Flood et al., 2014) for short dietary questionnaires validated for 2–5‐year‐old children. Overall, these results suggest that the EPOCH‐DQ is reliable for assessing diet in this population.

Interpretation analysis using cross‐classification revealed more than two‐thirds of participants were classified into the same risk categories when comparing the EPOCH‐DQ to the InFANT FFQ (i.e., validity), with stronger agreement between EPOCH‐DQ administrations (i.e., reliability). Overall, the 82%–84% average agreement in classifications exceeds previous short dietary questionnaires, whereby three‐quarters of participants aged 12–36 months were classified into the same risk category between administrations (Bell et al., 2014).

Although short tools are useful for screening, monitoring and evaluation purposes, short tool development is challenged by the need to prioritise and select items for inclusion. In this study, an item pool was developed and refined through a prioritisation and consensus process with experts in dietary assessment and early childhood obesity prevention. Nonetheless, further refinement of the EPOCH‐DQ is possible to enhance its ability to accurately and reliability assess obesity‐related diet behaviours in young children in practice. For example, future refinements may include reducing the number of items further. That is, the number of vegetables included in the vegetable variety question could be reduced by excluding those vegetables consumed less frequently and those that performed poorly, according to Cronbach's *α*. From the diet risk questions, pizza was infrequently consumed and performed poorly in the validation, so consideration could be given to removing this item from the scale. However, adaptations to the diet risk item score based on Cronbach's *α* should consider the limited number of items presently included. Further, due to variations in dietary intake across population groups, the performance of the EPOCH‐DQ should be evaluated for all ethnicities and ages (Golley et al., 2017).

These findings should be interpreted in the context of the study's strengths and limitations. The EPOCH‐DQ has been developed with multiple sources of population‐specific evidence and considers national public health recommendations. The questionnaire is simple to administer, taking only 5–10 min to complete. Furthermore, the development process of the EPOCH‐DQ was comprehensive, and versions were specifically developed to consider dietary risks at various stages of early childhood using a nationwide sample. Nonetheless, the study findings should consider that the comparison InFANT FFQ was designed for use in children aged 1.5–5 years and has not been validated for use in children aged 6–18 months (Zheng et al., 2020). Both the InFANT FFQ and EPOCH‐DQ are parent‐administered questionnaires which have the potential for social desirability bias (Hebert et al., 1995). As a result, these biases are likely to correlate across the two instruments and have the potential to inflate reliability and validity performance (Kirkpatrick et al., 2019). However, the use of a variety of statistical tests in the study design and assessment of validity should negate this limitation. Furthermore, the EPOCH‐DQ was developed through a rigorous process to determine content and structural validity before extensive statistical analysis of concurrent validity, construct validity, internal consistency, interpretability and test–retest reliability were completed. However, it must be acknowledged that the low prevalence of intake of some diet risk items violated the assumptions of *κ* and *χ* ^2^ tests.

In summary, the EPOCH‐DQ is a short assessment tool to identify dietary risk behaviours in early childhood. The present study concludes that the EPOCH‐DQ shows acceptable reliability and validity. The low participant burden and short length of the EPOCH‐DQ allow for potential applications in various settings. This includes use within the research setting for population health monitoring of children's dietary risk and use by health professionals to rapidly assess the dietary behaviours of young children and identify those at risk, allowing for intervention to improve dietary habits. Future development of the EPOCH‐DQ should include evaluation of results across culturally and socio‐economically diverse populations. Additionally, research is needed to establish the predictive validity and sensitivity to detect changes in the EPOCH‐DQ.

## AUTHOR CONTRIBUTIONS

Dorota Zarnowiecki, Lucinda Bell, Rebecca Byrne, Rachael Taylor, Li Ming Wen, Miaobing Zheng and Rebecca Golley designed the study. Dorota Zarnowiecki, Alexandra Manson, Shi Ning Tan and Rebecca Golley completed the data collection. Dorota Zarnowiecki, Alexandra Manson and Shi Ning Tan performed the data analysis. All authors interpreted the findings. Dorota Zarnowiecki, Alexandra Manson and Shi Ning Tan drafted the manuscript. All authors contributed to editing and had final approval of the submitted and published versions.

## CONFLICT OF INTEREST STATEMENT

The authors declare no conflict of interest.

## Supporting information

Supporting information.

## Data Availability

Data that support the findings of this study are available from the corresponding author upon reasonable request. The EPOCH‐DQ is available from www.flinders.edu.au/caring-futures-institute/dietary-assessment-tools.
