# Supplementary material for: Development and validation of a short dietary questionnaire for assessing obesity‐related dietary behaviours in young children
Source: Matern Child Nutr. 2024 Jan 8;20(2):e13613. doi: 10.1111/mcn.13613 (PMC10981485; doi:10.1111/mcn.13613)
Supplement: Supplementary file 1 — Supporting information. [file MCN-20-e13613-s001.docx]

**Supplementary Files**

**Table S1** Percentage of participants classified into the same category between questionnaire administrations

|  | Study 1 | | Study 2 | |
| --- | --- | --- | --- | --- |
|  | **T1 and InFANT**  (*n*=113) | **T1 and T2**  (*n*=70) | **T1 and InFANT**  (*n*=96) | **T1 and T2**  (*n*=55) |
| **Diet quality items** |  |  |  |  |
| Vegetable |  |  |  |  |
| *Number of categories*† | 67.3 | 74.3 | 79.2 | 80.8 |
| *Dark green* | 86.7 | 81.4 | 81.3 | 84.6 |
| *Orange* | 92.0 | 91.4 | 94.8 | 96.2 |
| *Salad* | 87.6 | 70.0 | 89.6 | 88.5 |
| *Starchy* | 81.4 | 78.6 | 88.5 | 86.5 |
| *Legumes* | 75.2 | 71.4 | 83.3 | 69.2 |
| *Other* | 85.8 | 82.9 | 84.4 | 88.5 |
| Vegetable Frequency | 83.2 | 88.6 | 62.5 | 82.7 |
| Bread type | 74.3 | 81.4 | 81.3 | 92.3 |
| Dairy type | - | - | 92.7 | 71.2 |
| **Diet risk score** |  |  |  |  |
| Sweetened Beverage | 95.6 | 97.1 | 85.4 | 86.5 |
| Non-core foods | 68.1 | 84.3 | 61.5 | 90.4 |
| **Feeding practices** |  |  |  |  |
| Bottle finished | - | 91.4 | - | - |

†score (0-6)

**Table S2** Vegetable classification, according to the Eat for Health Educators Guide^12^

| Vegetable group | Educators guide classification | EPOCH-DQ items | InFANT FFQ items |
| --- | --- | --- | --- |
| Dark green or cruciferous vegetables | Bok choy  Spinach  Broccoli  Cauliflower  Cabbage  Brussels sprouts | Asian greens (i.e. boy choy)  Spinach, baby spinach, rocket & other leafy greens  Lettuce  Asparagus  Celery  Broccoli  Cauliflower  Brussel Sprouts  Cabbage | Broccoli  Cauliflower |
| Orange vegetables | Sweet potato  Pumpkin  Carrots | Carrots  Sweet potato  Pumpkin | Carrots  Pumpkin |
| Salad vegetables | Lettuce  Tomato  Cucumber  Capsicum | Cucumber  Capsicum  Tomato | Cucumber  Capsicum  Tomato |
| Starchy vegetables | Potatoes  Sweet potato  Taro Corn | Potato  Sweet potato  Sweetcorn | Potato  Corn |
| Legumes | Dried peas  Beans  Lentils  Chickpeas | Peas, beans, snow peas, snap peas  Legumes (i.e. chickpeas, lentils, kidney beans) | Peas |
| Other | All other | Avocado  Eggplant  Mushrooms  Zucchini  Vegetables in mixed dishes (soups & stews)  Mixed frozen vegetables  Other (e.g. olives, onions, beetroot, radish) | Avocado  Mushrooms  Zucchini  Onion |

**Table S3** Percentage agreement of vegetable items between EPOCH-DQ administrations

|  | Study 1  (6-12 months) | | | | Study 2  (1-5 years) | | | |
| --- | --- | --- | --- | --- | --- | --- | --- | --- |
| **Food Items** | **Frequency (n)** | | **Kappa** | **% of agreement** | **Frequency (n)** | | **Kappa** | **% of agreement** |
|  | **T1** | **T2** |  |  | **T1** | **T2** |  |  |
| **Asparagus** | - | - | - | - | 4 | 4 | 0.73 | 96.4 |
| **Cabbage** | - | - | - | - | 5 | 4 | 0.64 | 94.6 |
| **Asian greens (i.e. boy choy)** | - | - | - | - | 2 | 4 | 0.30 | 92.9 |
| **Carrot** | 61 | 58 | 0.39 | 84.3 | 49 | 50 | 0.57 | 91.1 |
| **Cucumber** | 25 | 23 | 0.75 | 88.6 | 29 | 28 | 0.82 | 91.1 |
| **Eggplant** | - | - | - | - | 6 | 2 | 0.21 | 89.3 |
| **Brussels Sprouts** | - | - | - | - | 4 | 2 | -0.05 | 89.3 |
| **Avocado** | 45 | 39 | 0.41 | 71.4 | 28 | 27 | 0.75 | 87.5 |
| **Lettuce** | - | - | - | - | 14 | 12 | 0.60 | 85.7 |
| **Mushroom** | 47 | 44 | 0.6 | 84.3 | 22 | 18 | 0.69 | 85.7 |
| **Cauliflower** | 18 | 19 | 0.55 | 78.6 | 23 | 22 | 0.67 | 83.9 |
| **Celery** | - | - | - | - | 14 | 7 | 0.49 | 83.9 |
| **Broccoli** | 57 | 44 | 0.28 | 70.0 | 36 | 37 | 0.65 | 83.9 |
| **Legumes (i.e. chickpeas, lentils, kidney beans)** | 32 | 31 | 0.62 | 81.4 | 21 | 20 | 0.65 | 83.9 |
| **Tomato** | 38 | 38 | 0.54 | 77.1 | 36 | 34 | 0.62 | 82.1 |
| **Mixed frozen vegetables** | 7 | 7 | 0.37 | 88.6 | 17 | 13 | 0.55 | 82.1 |
| **Spinach, baby spinach, rocket & other leafy greens** | 35 | 32 | 0.51 | 75.7 | 28 | 25 | 0.61 | 80.4 |
| **Peas, beans, snow peas, snap peas** | 47 | 44 | 0.72 | 87.1 | 48 | 40 | 0.38 | 78.6 |
| **Corn, baby corn** | 35 | 32 | 0.57 | 78.6 | 43 | 45 | 0.37 | 78.6 |
| **Capsicum** | 24 | 25 | 0.59 | 81.4 | 24 | 20 | 0.55 | 78.6 |
| **Potato (baked or boiled, not fried)** | 44 | 47 | 0.47 | 75.7 | 40 | 37 | 0.46 | 76.8 |
| **Pumpkin** | 53 | 48 | 0.33 | 72.9 | 30 | 25 | 0.54 | 76.8 |
| **Zucchini** | 42 | 37 | 0.51 | 75.7 | 33 | 26 | 0.54 | 76.8 |
| **Sweet Potato** | 53 | 50 | 0.23 | 70.0 | 29 | 26 | 0.47 | 73.2 |
| **Other (e.g. olives, onions, beetroot, radish)** | 40 | 40 | 0.65 | 82.9 | 25 | 19 | 0.41 | 71.4 |
| **Vegetables in mixed dishes (soups & stews)** | 35 | 28 | 0.51 | 75.7 | 37 | 26 | 0.34 | 66.1 |

‑

^1^ Byrne, R., et al (2018) Brief tools to measure obesity-related behaviours in children under 5 years of age: a systematic review. *Obesity Reviews* https://doi.org/10.1111/obr.12801

^2^ Zarnowiecki, D., et al (2020) Improving the reporting of young children’s food intake: Insights from a cognitive interviewing study with mothers of 3-7-year old children *Nutrients*<https://doi.org/10.3390/nu12061645>

**Figure S1** Development process of the EPOCH-DQ


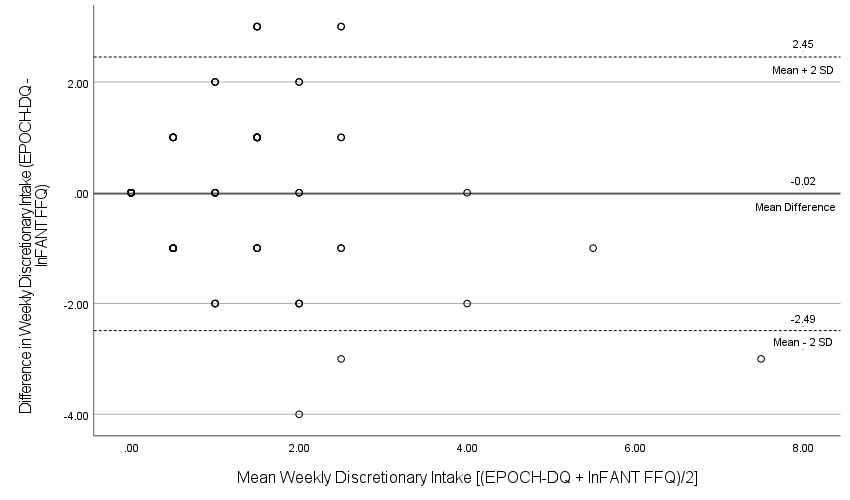


**Figure S2** Bland-Altman plot visualising the agreement between weekly discretionary food intake computed from Study 1, comparing the infant version EPOCH-DQ items and the InFANT FFQ.

‒‒ represents the mean difference between the EPOCH-DQ and InFANT FFQ; ---- represents the limits of agreement


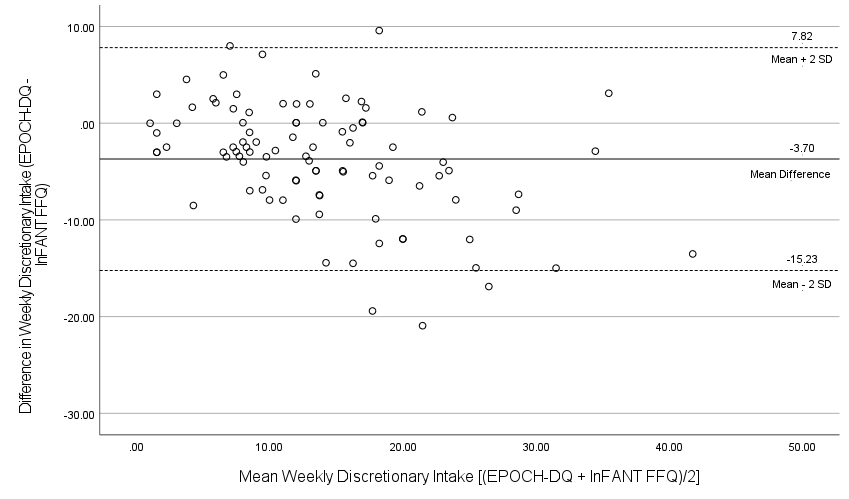


**Figure S3** Bland-Altman plot visualising the agreement between weekly discretionary food intake computed from Study 2, comparing the 1–5-year-old versions of the EPOCH-DQ and the InFANT FFQ.

‒‒ represents the mean difference between the EPOCH-DQ and InFANT FFQ; ---- represents the limits of agreement
